# Should community health workers offer support healthcare services to survivors of sexual violence? a systematic review

**DOI:** 10.1186/s12914-017-0137-z

**Published:** 2017-10-12

**Authors:** Anne Gatuguta, Barbra Katusiime, Janet Seeley, Manuela Colombini, Isaac Mwanzo, Karen Devries

**Affiliations:** 1 Department of Global Health and Development, Faculty of Public Health and Policy, London School of Hygiene and Tropical Medicine, Keppel Street, London, WC1E 7HT UK; 2 Department of Community Health, School of Public Health, Kenyatta University, Nairobi, Kenya; 3 Mbarara University of Science & Technology, Mbarara, Uganda; 4 Department of Pharmacy, Kent University, Kent, UK

**Keywords:** Sexual violence, Community health workers, Sexual violence healthcare services, Survivors

## Abstract

**Background:**

Sexual violence is widespread, yet relatively few survivors receive healthcare or complete treatment. In low and middle-income countries, community health workers (CHWs) have the potential to provide support services to large numbers of survivors. The aim of this review was to document the role of CHWs in sexual violence services. We aimed to: 1) describe existing models of CHWs services including characteristics of CHWs, services delivered and populations served; 2) explore acceptability of CHWs’ services to survivors and feasibility of delivering such services; and 3) document the benefits and challenges of CHW-provided sexual violence services.

**Methods:**

Quantitative and qualitative studies reporting on CHWs and other community-level paraprofessional volunteer services for sexual violence were eligible for inclusion. CHWs and sexual violence were defined according to WHO criteria. The review was conducted according to the Preferred Reporting Items for Systematic reviews and Meta-Analyses guidelines. Quality of included studies was assessed using two quality assessment tools for quantitative, and, the methodology checklist by the National Institute for Health and Clinical Excellence for qualitative studies. Data were extracted and analysed separately for quantitative and qualitative studies and results integrated using a framework approach.

**Results:**

Seven studies conducted in six countries (Democratic Republic of Congo, Rwanda, Burma, United States of America, Scotland, Israel) met the inclusion criteria. Different models of care had diverse CHWs roles including awareness creation, identifying, educating and building relationships with survivors, psychosocial support and follow up. Although sociocultural factors may influence CHWs’ performance and willingness of survivors to use their services, studies often did not report on CHWs characteristics. Few studies assessed acceptability of CHWs’ to survivors or feasibility of delivery of services. However, participants mentioned a range of benefits including decreased incidence of violence, CHWs being trusted, approachable, non-judgmental and compassionate. Challenges identified were high workload, confidentiality issues and community norms influencing performance.

**Conclusions:**

There is a dearth of research on CHWs services for sexual violence. Findings suggest that involving CHWs may be beneficial, but potential challenges and harms related to CHW-provided services exist. No different models of CHW-provided care have been robustly evaluated for effects on patient outcomes. Further research to establish survivors’ views on these services, and, their effectiveness is desperately needed.

**Electronic supplementary material:**

The online version of this article (10.1186/s12914-017-0137-z) contains supplementary material, which is available to authorized users.

## Background

Sexual violence is widespread globally. Reported lifetime prevalence of partner and non-partner sexual violence in women is as high as 59% and 12% respectively in some regions [1–3]. Associated health consequences are both short-term and long-term [1, 4–10]. Global clinical guidelines recommend comprehensive immediate healthcare and follow up to address both the clinical and psychosocial needs of survivors [11–15]. However, the majority of survivors do not access health care and only a limited proportion complete recommended treatment [16–25]. Sexual violence stigma at the community level, distance from health facilities, unreliable or unavailable services, healthcare professionals’ attitudes and competing priorities for survivors such as work are some of the main barriers to access [25–27]. Lack of active follow up and social support further hinder treatment completion [18, 25]. Moreover, most services are provided through emergency care or rape care centres, where fear of stigma and being judged may prevent participants from attending [18]. One way to overcome the poor access to healthcare and to treatment completion is to make use of already existing and affordable structures such as community health worker (CHW) services [28, 29].

The World Health Organisation (WHO) defines CHWs as community-based workers who are members of the communities where they work, selected by their communities, have received limited training but are not professional health workers. They are supported by the health system while not necessarily being a part of its organisation [30]. Community health workers provide a means for communities to access affordable healthcare as well as participate in managing their health [31]. Studies show that CHWs can provide benefits in cost-savings, increasing community involvement, improving clinical outcomes, providing an alternative to professional workforce-limited situations and decongesting health facilities [32].

There have been concerted efforts, particularly in resource-limited settings, to utilise CHWs in the management of different health conditions. Data show the positive impact of CHWs on access to care, clinical, retention and other outcomes in treatment of specific health conditions such as maternal and child health [33], tuberculosis (TB) [34], human immunodeficiency virus/acquired immune deficiency syndrome (HIV/AIDS) [32, 34, 35] and mental health [36, 37]. A systematic review of community-based HIV treatment in Sub-Saharan Africa for instance, showed that interventions delivered by CHWs could reduce barriers to retention and reduce costs to patients [38].

In this paper we report the findings of a review to investigate whether CHWs could provide similar benefits in sexual violence healthcare for adults and children. We reviewed different components of CHW programmes that could potentially influence CHW performance as well as interactions with survivors. These components included the socio-demographic characteristics of CHWs used, their selection, training provided, roles of the CHWs, mode of service delivery and population served. The review also assessed the acceptability of CHWs services by survivors, the feasibility of delivering such services, as well as the challenges and benefits associated with such services for CHWs, health care systems and survivors.

## Methods

We conducted a systematic review of qualitative and quantitative studies. The review was conducted and reported according to the Preferred Reporting Items for Systematic reviews and Meta-Analyses (PRISMA) guidelines [39] and was registered with the International Prospective Register of Systematic Reviews (PROSPERO).

Inclusion criteria:

All studies that reported on services or interventions delivered by CHWs to support prevention of sexual violence, access to healthcare, treatment adherence and retention in healthcare for sexual violence survivors were included. Community health workers were defined based on the WHO definition [30]. Many of the studies referred to these workers as volunteers and therefore the term CHWs and volunteers are used here interchangeably. Sexual violence was defined based on the WHO definition as “any sexual act, attempt to obtain a sexual act, unwanted sexual comments or advances, or acts to traffic, or otherwise directed, against a person’s sexuality using coercion, by any person regardless of their relationship to the victim, in any setting, including but not limited to home and work” [40].

### Search strategy

Seven databases were searched from first record to 17/05/2017: MEDLINE, Africa Wide Information, Cumulative Index to Nursing and Allied Health Literature (CINAHL) Plus, Cochrane library, Embase, Global Health and PsychINFO. References of identified studies were also checked for relevant studies. There was no time or language restriction to the studies. A search criteria tailored for each database was developed with the relevant controlled vocabulary terms, Boolean operators and truncation applied to the different databases. The search terms for CHWs included community health worker*, CHW*, lay health worker*, community own resource person*, CORP*, patient advocate*, close to community health worker*, community health aide*, and village health worker. The search terms for sexual violence included sexual violence, sexual abuse, sexual molestation, sexual assault, rape, date rape, defilement, incest, sodomy, child sexual abuse, post exposure prophylaxis, PEP, ARV*, antiretroviral*, HIV PEP, nPEP and n-PEP. The search strategy and number of articles obtained is outlined in Additional file 1: Appendix 1.

**Fig. 1:**
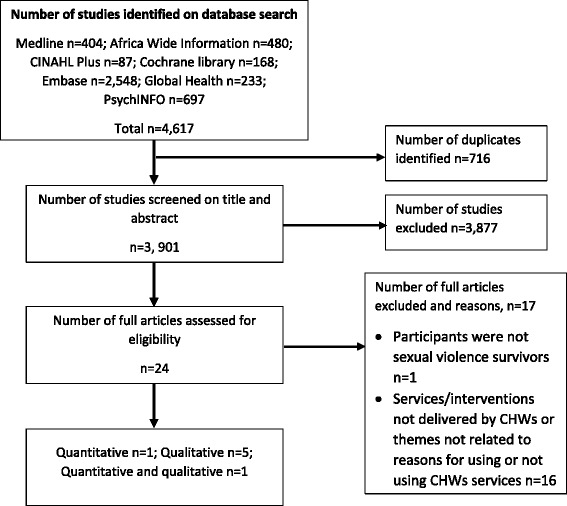
Literature search results

In total, 4617 records were obtained, saved into an EndNote X7 library and duplicates removed. The remaining 3901 records were screened on titles and abstracts and irrelevant studies identified excluded from further screening. Full texts were obtained for 24 studies. A further 17 studies were excluded based on the full text review due to various reasons as shown (Fig. 1). All the titles and abstracts for inclusion were screened by AG; AG and BK read and screened the 24 full text studies.

### Data abstraction

All information related to supporting prevention, access to care, treatment and retention was extracted. Acceptability and feasibility were assessed through reported willingness to use, satisfaction with services, ease of delivery, quality and uptake of services, availability of resources, adequacy of training and cost-effectiveness of services. Additionally, data extracted included: the type of study design, study setting, participants socio-demographic characteristics, number of participants, study methods including recruitment and retention, outcomes measured and results.

For qualitative studies, all data and themes identified by the author in the paper were abstracted [41]. As our study is exploratory, we took this more inclusive approach to allow more data and themes to be included for analysis [41]. Data were abstracted on data collection methods used including number and composition of group members, consenting process, data processing and analysis, identified themes and findings from the study.

### Methodological quality of included studies

Quantitative and qualitative studies were assessed separately for methodological quality. The quality of the studies was considered critically and findings are interpreted in light of this, however, no studies were excluded based on their quality. As the two quantitative studies were vastly different in design (one a longitudinal descriptive study and the other a pre and post-test design) two quality assessment tools were used. Quality assessment tool for quantitative studies developed by the Effective Public Health Practice Project (EPHPP) was used for the pre and post-test study [42] while quality assessment checklist for observational studies (QATSO Score) was used for the longitudinal study [43]. An overall rating of the whole study is normally constituted from all quality domains; however the Cochrane collaboration discourages the assigning of a summary score as this involves assigning weights to different domains which may not be justifiable [44]. For this reason, both quantitative studies were rated on each domain but an overall quality score was not constituted (Additional file 1: Appendix 2). Quality assessment for the qualitative studies was done using the methodology checklist for qualitative studies by the National Institute for Health and Clinical Excellence (NICE) [45] and is shown in Additional file 1: Appendix 3.

### Bias

Due to limited time and resources, a comprehensive search for grey literature was not done, so some studies may not have been included. Nevertheless, publication bias was minimised by searching multiple databases as reported, and although not comprehensively done, Google Scholar, the London School of Hygiene and Tropical Medicine Research Online and the WHO’s website were also searched. Too few studies using the same outcome measures met the inclusion criteria to quantitatively assess publication bias. We did not find multiple articles reporting on the same study. No studies were excluded based on language and no time period was applied to the search.

### Data analysis and synthesis

Qualitative and quantitative data were analysed separately and the findings combined into a final synthesis. The two quantitative studies reviewed had different aims and outcomes, therefore a descriptive synthesis was performed in lieu of a meta-analysis. For qualitative studies, data were analysed thematically. Key themes relevant to the review questions were identified prior to data abstraction. Other relevant themes emerging from the review process were integrated in the analysis.

Data synthesis from both qualitative and quantitative studies was done through a framework approach [46] and summarised in matrices as illustrated in Table 1. The findings were summarised under the following headings: models of CHW services for sexual violence care, acceptability and feasibility of CHW services in sexual violence care and the challenges and benefits of CHWs as service providers for sexual violence.

**Table 1:** Representative matrix chart of data synthesis

Theme	Kohli, A. et al., 2012.	Tanabe, M. et al., 2013.	Barron, I. G. & Topping, K. J. 2013.	Merkin, L. & Smith, M. J. 1995.	Rossman, L. & Dunnuck, C. 1999.	Zraly, M., Rubin-Smith, J. & Betancourt, T. 2011.	Itzhaky, H. & York, A. S. 2001.
Acceptability of CHW services	Patients reported satisfaction with services provided; Few adolescents attended to, interviews with some revealed discomfort being seen in the same clinic with older women as being identified as SV survivor may diminish marriage opportunities	Community members reported that CHWs are trusted members of society that survivors can seek care from	Survivors reported liking the programme & the programme being understandable	Rising invitations to give lectures & workshops to the community	Rise in the use of volunteer advocates by 75%; feedback from victims of non-judgemental compassionate support provided	Women found the services useful and particularly when hospital services were inadequate for their needs	Feeling of trust for community workers developed; Large number of community members becoming involved in the prevention efforts
Feasibility of CHW services	Overall, the mobile clinic utilised limited human resources, equipment & medication	CHWs demonstrated comfort with the subject of sexual assault and good understanding of medical treatment; CHWs also demonstrated full understanding of confidentiality and data collection; Safety was not an issue of concern to CHWs	Cost of delivery was minimal particularly because the facilitators were volunteers. Training & experience contributed to facilitators spending very little time on preparation, one hour	No assessment of feasibility documented	No assessment of feasibility documented	Study identifies the potential opportunity to incorporate the current informal support networks for survivors with the national CHW programme being implemented	No assessment of feasibility documented

## Results

### Characteristics of included studies

Seven studies met the inclusion criteria [47–53]. The studies were conducted in six countries: Democratic Republic of Congo, Rwanda, Burma, United States of America, Scotland and Israel. Of those included, five were qualitative [48, 50–53], one quantitative [49] and one used both quantitative and qualitative methods [47]. Of the five qualitative studies, one was implemented for prevention of child sexual abuse in the community [48], the second described a volunteer advocate support programme for a specific population of the deaf and deaf-blind [50]. The third study described a stand-alone treatment centre for sexual violence which utilised volunteers to provide psychosocial support to survivors [51]. The fourth study described a pilot programme for community-based medical care for survivors delivered by CHWs [52] while the fifth comprised of 44 semi-structured interviews with survivors who were members of local survivors associations where select members were trained to provide trauma counselling to their peers [53].

The quantitative study was a longitudinal descriptive study that followed up survivors over a one month period and utilized CHWs attached to a mobile clinic at the community level [49]. The mixed methods study involved a pre- and post-test waitlist design with a volunteer-delivered prevention programme. Quantitative methods were used to compare pre- and post-intervention knowledge, skills and occurrence of violence disclosure while qualitative methods were used to assess acceptability and feasibility of the programme [47]. The characteristics of the studies are summarised in Table 2.

**Table 2:** Summary of characteristics of included studies

Author, Year	Country	Study design	Number of survivors	Age of survivors	Socio-demographics characteristics of survivors	Type of services received by survivors	Community health workers (CHWs) service model	Number of community health workers
Kohli, 2012 [49]	Democratic Republic of Congo (DRC)	Observational: Longitudinal follow-up for one month	657 survivors received medical treatment	0.9% below 20 years, 59.6% above 40 years	Females, 3.7% single, 61.9% married, 19.8% separated & 14.6% widowed	Treatment for sexually transmitted infections (STIs) and other diseases, HIV testing, psychosocial support	General CHWs attached to a mobile clinic	Not reported
Tanabe, 2013 [52]	Burma	Qualitative: Focus group discussions with CHWs, traditional birth attendants & community members	No survivor presented	No survivor presented	No survivor presented	Medical treatment of STIs, pregnancy prevention, wound care, psychosocial support & referral	Specialised CHWs providing mobile maternal health care at the community level & trained to provide sexual violence care	Not reported
Barron, 2013 [47]	Scotland	Mixed methods: Experimental pre- & post-test design; Qualitative in-depth interviews	20 included in the study	6–13 years	Intervention group: 4 males, 6 females; comparison group 10 females	Small group training- 4 lessons of 50 min duration on child sexual abuse prevention	^a^Volunteer workers trained in delivering the programme	3 females
Merkin, 1995 [50]	United States of America	^b^ Qualitative: Observations, informal conversations	225 cases	4–76 years: (205 adults & 20 children)	204 females & 21 males; 197 deaf & 28 deaf-blind	Crisis intervention, medico-legal & social support	^a^Volunteer workers trained in gender-based violence & in supporting survivors	18 females & 2 males
Rossman, 1999 [51]	United States of America	^b^ Qualitative: Observations, informal conversations	Not reported	Not reported	Not reported	Psychosocial support	^a^Volunteer workers attached to a community treatment centre	Not reported
Zraly, 2011 [53]	Rwanda	Qualitative semi-structured interviews	44 interviewees	Not reported	Females	Individual & group counselling	^a^Peer survivor trained in counselling	One female
Itzhaky, 2001 [48]	Israel	Qualitative in-depth interviews & observations	15 child sexual abuse cases identified	Children, age not reported	Children	Counselling	^a^Volunteer community workers	Not reported

^a^Volunteers trained to deliver the specific programme but not typical community health workers

^b^Studies did not describe a data collection method such as in-depth interviews or focus group discussions

### Methodological quality of quantitative studies

Only two quantitative studies were identified which met our inclusion criteria. One was a longitudinal descriptive study of survivors receiving medical treatment [49]; the other was a small pilot test of a prevention intervention with only 20 participants, which used a pre and post-test design [47]. The quality of the studies is summarised in Table 3. These are some of the first studies conducted on this topic and provide valuable information about how participants interact with services. However, the sample size was very small in the pre-post study, and attrition was extremely high in the longitudinal study (perhaps not surprisingly given that it was conducted in a conflict setting). No studies were found that intended to evaluate the effects of a CHW led intervention on survivor outcomes.

**Table 3:** Methodological quality rating of quantitative studies

Study	Rating domain (EPHPP for evaluation studies)							
	Selection bias	Study design	Confounders	Blinding	Data collection methods	Withdrawals and dropouts	Intervention integrity	Analyses
Barron, 2013 [47]	Moderate	Moderate	Weak	Not applicable	Moderate	Strong	Moderate	Moderate
	Rating domain (QUATSO for)							
	External validity		Reporting		Confounding		Bias (Privacy)	
Kohli, 2012 [49]	0		Response rate	Outcome measure	Not applicable		1	
			Not applicable	1				

### Methodological quality of qualitative studies

Three of the qualitative studies were scored as having met most of the checklist criteria (++) [47, 52, 53], one was scored as having met some of the criteria (+) [48] and two were scored as having met few of the criteria (−) [50, 51]. The quality assessment was limited by a lack of detail about reported methods, particularly in regards to data collection and analysis. The quality assessment for all the qualitative studies is summarised in Table 4.

**Table 4:** Methodological quality assessment for qualitative studies

Rating section	Barron, 2013 [47]	Itzhaky, 2001 [48]	Merkin, 1995 [50]	Rossman, 1999 [51]	Tanabe, 2013 [52]	Zraly, 2011 [53]
1.1 Is a qualitative approach appropriate?	Appropriate	Appropriate	Appropriate	Appropriate	Appropriate	Appropriate
1.2 Is the study clear in what it seeks to do?	Clear	Mixed	Mixed	Unclear	Clear	Mixed
2.1 How defensible/rigorous is the research design/methodology?	Defensible	Defensible	Not defensible	Not defensible	Defensible	Defensible
3.1 How well was the data collection carried out?	Appropriate	Appropriate	Inadequately reported	Inadequately reported	Appropriate	Appropriate
4.1 Is the context clearly described?	Clear	Clear	Unclear	Unclear	Clear	Clear
4.2 Were the methods reliable?	Reliable	Reliable	Unreliable	Unreliable	Unreliable	Unreliable
5.1 Are the data ‘rich’?	Rich	Not sure/not reported	Not reported	Not reported	Rich	Rich
5.2 Is the analysis reliable?	Reliable	Not reported	Not reported	Not reported	Unreliable	Reliable
5.3 Are the findings convincing?	Convincing	Not convincing	Not convincing	Convincing	Convincing	Convincing
5.4 Are the conclusions adequate?	Adequate	Adequate	Adequate	Adequate	Adequate	Adequate
6.1 Was the study approved by an ethics committee?	Yes	Not reported	Not reported	Not reported	Yes	Yes
6.2 Is the role of the researcher clearly described?	Clear	Not clear	Not clear	Not reported	Clear	Clear
As far as can be ascertained from the paper, how well was the study conducted?	++	+	–	–	++	++

### Components of CHW models for sexual violence services

The components of CHW models reported include the socio-demographic characteristics of CHWs, their selection, training, roles, mode of service delivery and population served (Table 5). In general, CHW services were delivered by volunteers trained on the specific intervention. Most were specialised volunteers who only delivered one type of service/intervention and there was no evidence that they carried out any other health-related activities [47, 50, 51, 53]. In nearly all cases, the volunteer programmes were run by non-governmental organisations (NGOs) but delivered services in collaboration with government agencies.

**Table 5:** Summary of characteristics of different models of CHWs services for sexual violence

	Socio-demographic characteristics of CHWs	Selection of CHWs	Training of CHWs	Roles CHWs	Mode of service delivery	Population served
Kohli, 2012 [49] Democratic Republic of Congo	Not documented	Respected community members known for supporting neighbours to deal with loss of family, rejection and stigma due to sexual violence.	Training in the provision of ethical, compassionate and competent care for GBV survivors.	Identify & build relationships with survivors & educate them on available services; assist providers in prioritising services; spread the word throughout the village about the mobile clinic visit schedule & encourage women and others to attend	General CHWs attached to a mobile clinic	All survivors of sexual violence
Tanabe, 2013 [52] Burma	Not documented	Highest cadre of CHWs- previously trained to provide reproductive health services	5 days training on care of SV survivors, 3 day refresher training every 6 months	Conducting medical examination, treatment or preventive treatment for STIs, emergency contraception, care of wounds, supportive counselling & referral	Specialised CHWs providing mobile maternal health care at the community level	No survivors presented during the study period
Barron, 2013 [47] Scotland	3 Female; 20–30 years; working class; adult survivors of child sexual abuse	Not documented	Trained & experienced in delivering the programme- training details not given	Facilitate small group training sessions	Volunteer workers delivering group training	Children
Merkin, 1995 [50] United States of America	18 female & 2 male	Rigorous screening including an interview to determine suitability	50-h training on gender-based violence & programme delivery; continuous monthly meetings & trainings	Receive calls on the crisis line, educating victims, accompany victims to hospital & police	Volunteer workers attached to a crisis centre	All survivors both male and female who are deaf & deaf-blind
Rossman, 1999 [51] United States of America	Not documented	Not documented	Not documented	Setting up counselling appointments; provide emotional support to the victims	Volunteer advocates attached to a community treatment centre	Not documented
Zraly, 2011 [53] Rwanda	Female	Not documented	12 weeks theory & 250 h of supervised practice on trauma counselling	Providing individual & group counselling; assisting with setting up peer support associations	Members of survivor support associations trained to provide counselling	Adult female
Itzhaky, 2001 [48] Israel	Not documented	Not documented	Workshop-type training on child abuse & incest	Identified cases of abused children, conducted community awareness and group trainings	Community workers/activists not affiliated to the health system	Children

#### Socio-demographic characteristics of CHWs used

Only 3 of 7 studies reported any socio-demographic characteristics of the CHWs. One study reported using only female CHWs aged between 20 and 30 years [47] while another reported using both females and gay men to serve gay male survivors [50]. A third study described using peers who were female [53]. Even among the studies that reported this information, none reported how these characteristics may or may not have affected the outcomes measured or relationships with survivors.

#### Selection of CHWs

The selection of the CHWs was reported in 3 of 7 studies. Where reported, the selection appeared to target CHWs with specific skills suited to serving survivors. One study reported selecting the ‘highest cadre’ of CHWs, defined as CHWs who had previously been trained to provide reproductive health services [52]. These CHWs were therefore deemed already equipped with the skills they needed, for instance to provide emergency contraception to survivors. A second study reported screening volunteers for their ability to understand the importance of confidentiality and sensitivity [50]. A third study described selecting respected community members already known for supporting individuals dealing with grief, rejection and sexual violence stigma [49].

#### Training of CHWs

The training details for the CHWs were not given in two studies [47, 51]. In the other five studies, the duration of training varied broadly, and there was a lack of detail about the content of training curricula. One study reported a ‘workshop type’ training on child abuse and incest [48]. The training materials included topics on child abuse, dynamics of abusing families, societal attitudes towards child sexual abuse (CSA), how to communicate with victims and reporting of abuse among others. Two studies reported at least five days training with frequent refresher courses [50, 52]. The topics covered included medical care of survivors, psychosocial support and referral. The fourth study reported 12 weeks of theory and 250 h of practice on trauma counselling [53] while the fifth only mentioned providing training in the provision of ethical, compassionate and competent care for gender-based violence survivors [49]. None of the studies assessed the effect, duration or the contents of training on any outcomes related to survivors or CHWs.

#### Roles of the CHWs and mode of service delivery

Different modes of service delivery were reported. In some programmes, CHWs worked at the community level [48, 52, 53] while in others they were based in a facility [50, 51] and yet in others they were involved both at the community and facility [49]. For those based in the community, activities included raising awareness, identifying cases, treatment, providing community feedback to healthcare workers at health facilities and providing psychosocial support including individual and group counselling of survivors. Those based in facilities responded to crisis telephone calls, accompanied survivors to hospitals and the police, provided emotional support and education as well as assisted clinicians in tasks related to managing survivors such as prioritising treatment, setting up appointments and follow-up. One programme had volunteers providing group-training on knowledge and skills for prevention of child sexual abuse to children with previous exposure to violence. The role of treating survivors including wound care, prescribing treatment such as emergency contraception and prophylaxis for sexually transmitted infections was piloted by one study [52]. Although this pilot was carried out for one year and CHWs reported being comfortable providing these services, no survivor was treated and it is therefore difficult to draw any conclusions on the capacity of CHWs delivering medical care to survivors.

#### Populations served

With regards to populations served, the prevention services targeted prevention of violence in children with interventions delivered to both the community and groups of children [47, 48]. Services and interventions providing medical care and psychosocial support targeted adult women [49, 52]. In some instances, the services were provided as part of a support group formed by survivors and ran by peers [53]. One programme was designed exclusively for a specific population of the deaf and deaf-blind which included both males and females [50]. It is notable however, that the proportion of clients who were males served by this intervention was low (9.3%).

### Acceptability and feasibility of CHWs services in sexual violence care

Assessment of the acceptability and feasibility of CHW services was limited and equivocal as summarised in Table 6. Only three studies reported collecting data from survivors on their experience with CHWs services [47, 51, 53]. Survivors in one study found the services useful in improving access to care and providing psychosocial support particularly when hospital services were inadequate for their needs [53]. In the other two studies, programmes delivered by CHWs were reported as likeable and understandable as well as providing non-judgemental and compassionate support [47, 51]. These findings on acceptability are limited by the small number of survivors interviewed. It is also notable that the studies either did not assess or did not report any negative concerns from survivors.

**Table 6:** Reported acceptability and feasibility of CHWs in sexual violence services

	Acceptability	Feasibility
Kohli, 2012 [49]	Not documented- assessment of whole programme rather than CHWs	Not documented- assessment of whole programme rather than CHWs
Tanabe, 2013 [52]	Community members interviewed reported that CHWs are trusted members of society that survivors can seek care from	CHWs demonstrated comfort with the subject of sexual assault and good understanding of medical treatment; CHWs demonstrated full understanding of confidentiality and data collection; Safety was not an issue of excess concern to CHWs
Barron, 2013 [47]	Survivors reported liking the programme & the programme being understandable	Cost of delivery was minimal particularly because the facilitators were volunteers. Training & experience contributed to facilitators spending very little time on preparation
Merkin, 1995 [50]	Not documented- assessment of programme rather than CHWs	No assessment of feasibility documented
Rossman, 1999 [51]	Rise in the use of volunteer advocates by 75%; feedback from victims of non-judgemental compassionate support provided	No assessment of feasibility documented
Zraly, 2011 [53]	Interviewed women found the services useful and particularly when hospital services were inadequate for their needs	No assessment of feasibility documented
Itzhaky, 2001 [48]	Feeling of trust for community workers developed; Large number of community members becoming involved in the prevention efforts	No assessment of feasibility documented

Notably, only one of the studies in the review was designed specifically to assess the performance of CHWs in delivering healthcare services to survivors [52]. In this study, community members interviewed expressed the view that CHWs were trusted members of the community who could be approached by survivors for help. Although the study design included a plan to interview survivors, this did not happen as no survivor presented within the one year study period. The reasons why survivors did not present are not clear, with the authors suggesting possible reasons as no incidence occurring, sensitivity of the subject or lack of awareness of the existence of services. The other studies were designed to assess programmes in which CHWs were a component. As such, reported data focused more on the overall programme acceptability rather than the CHWs. Additionally, reports are mainly from other stakeholders’ perspectives and not the survivors themselves thus posing an impediment to the extent that acceptability of the services could be assessed.

Only two studies assessed any aspect of feasibility of sexual violence service delivery by CHWs. Interviewed CHWs reported being comfortable dealing with sexual violence, understanding the services they were to provide and the confidentiality required, and having no safety concerns [52]. The second study assessed the cost of delivering the programme and reported it to be minimal due to CHWs being volunteers therefore unpaid [47]. However, this was not a formal costing but an estimate of the cash used to deliver the programme. This study also reported limited preparation time for the CHWs once they had received initial training and gained experience.

### Challenges and benefits of CHWs providing services for sexual violence

Several benefits and challenges were mentioned (Table 7). Authors in two studies reported that enhanced community awareness and knowledge as a result of CHWs activities and advocacy resulted in fewer incidences of violence, increased number of survivors taking action to end violence from partners and an increased number of cases of abuse going to trial [48, 50]. Nevertheless, these studies were small and larger prospective studies with control groups are needed to assess the effect of similar interventions over time. Two studies reported feedback from survivors that they received non-judgemental, compassionate and useful psychosocial support from CHWs [51, 53]. Similarly, the authors of one study observed that the programme benefited by having CHWs among their cadre of staff [49]. The CHWs provided feedback that assisted healthcare providers to understand the local concerns which they could then address during health talks or individual treatment. They were also able to mobilise communities and survivors to access the available healthcare which they might have missed without the awareness created.

**Table 7:** Reported benefits and challenges of CHWs in sexual violence services

	Benefits	Challenges
Kohli, 2012 [49]	Authors report that local CHWs assisted healthcare providers in targeting education sessions to community concerns; CHWs provided feedback to healthcare providers e.g. reported increased patient satisfaction	Authors report “travel distance & other commitments sometimes prevented CHWs from reminding patients about appointments and thus, follow-up rates were not as high as expected.”
Tanabe, 2013 [52]	Community reported that CHWs are trusted persons that survivors can approach for help	CHWs reported lacking confidence in history-taking and psychosocial care; ‘Lower cadres’ of CHWs were unhappy with some aspects of medical care & referrals, complained they already had too many responsibilities, had issues with maintaining confidentiality & had some safety concerns
Barron, 2013 [47]	Increased knowledge & skill; Occurrence of disclosures in the intervention group compared to no disclosures in the comparison group; satisfaction with programme; minimal cost of delivery	Not documented
Merkin, 1995 [50]	Increased number of victims taking action on violence in their lives & increase in number of cases of abuse going to trial	Not documented
Rossman, 1999 [51]	Feedback from victims report non-judgemental compassionate support by volunteers	Time taken to contact the volunteer & get them to the centre to offer support was long delaying care for survivors; Failure of recognition & acceptance by both the victim & professional healthcare workers
Zraly, 2011 [53]	Available care in crisis & source of support	Not documented
Itzhaky, 2001 [48]	Increased community awareness with change of attitude towards child sexual abuse; Reduction in stigma & therefore increased acceptance & support for survivors; Reduced incidence of cases	Child abuse reportedly normative thus community workers not motivated to act initially

There were general challenges related to CHWs providing sexual violence services and challenges related to specific models of CHWs. Where CHWs provided other health-related services (general CHWs), issues of many responsibilities and high workload were reported [49, 52]. In addition, other commitments that CHWs had, prevented them from delivering the services effectively and in some instances, CHWs were expected to cover vast regions hindering their ability to reach everyone [49].

With regards to sexual violence, some of the CHWs interviewed were uncomfortable with certain aspects of care, (for example, medical care) while others were unclear on how to assist survivors while maintaining confidentiality [52]. In one study, professional healthcare workers and survivors had a problem understanding the role of the CHWs which meant they were not always readily accepted as part of the care team [51]. Additionally, in programmes where CHWs were called in when a survivor presented, the time taken to contact the CHWs and get them to the facility was often long, therefore delaying care for the survivor [51]. One study reported concerns regarding community norms affecting the work of the CHWs who were themselves members of that community [48]. Child abuse was noted as being normative in this community and the CHWs also had difficulties recognising it as abuse.

## Discussion

To our knowledge, this is the first systematic review of CHW services for sexual violence against adults and children. This review has identified important gaps in research in this area. Firstly, there was no robust evidence to support any particular model or model components as being effective for the delivery of sexual violence services by CHWs. Secondly, evidence on the acceptability and feasibility of delivering these services is minimal in volume and generally limited in quality. Although there is some evidence suggesting that CHWs provide services for survivors, the experiences of the survivors themselves and CHWs with regards to these services are largely undocumented. Thirdly, in terms of benefits, the studies reviewed indicate a wide range of services provided by CHWs spanning from prevention, treatment, psychosocial support and follow up. However, it is unclear where CHWs provide the maximum benefit or where their knowledge and skills can be optimally utilised. Finally, various challenges were mentioned which highlight the importance of tailoring services to survivor needs, different populations and context and this area requires further exploration.

Our review had several limitations. Firstly, there were limitations related to the included studies: Few studies met the inclusion criteria. Very few survivors or CHWs were interviewed in these studies, therefore the studies mostly represent the views of other stakeholders rather than the actual consumers of services. It is therefore difficult to draw any conclusions on effectiveness of models, acceptability or feasibility of CHWs in sexual violence services. No studies evaluated the effects of any CHW-provided intervention to improve any aspect of sexual violence care. These limitations perhaps reflect the difficulties of conducting research in this topic and highlight the need for innovative ways to recruit and follow up survivors for research. More rigorous research in this area is necessary.

Secondly, there were limitations related to the review: While every effort was made to include as many studies as possible, the term community health worker represents a very broad concept with many different terms used to describe CHWs in different settings. It is possible that some of these terms were not included in our search strategy. We also included studies that did not define their volunteers as CHWs or volunteers who did not fit the typical WHO definition (for instance belonging to and being selected by the community) as long as they delivered similar services to CHWs. Additionally, our search terms for sexual violence did not include IPV. While we are confident that our search would have picked majority of the IPV studies reporting sexual IPV as an outcome, it is possible that we missed some studies reporting sexual IPV that was not disaggregated from other forms of IPV. Similarly, children were poorly represented among the studies reviewed. It is therefore possible that our findings particularly on the acceptability and feasibility of CHWs may differ among survivors of IPV and children.

Despite the limitations of the current evidence base, a number of important findings emerged. Some of the reviewed studies reported that CHWs were trusted members of the community and this trust can enhance their role in awareness raising and mobilisation. This trusted position has enabled CHWs to successfully provide services for other health conditions such as maternal and child health, HIV, TB, malaria, and mental health [28]. Trust is likely to be of key importance for sexual violence, a stigmatised condition; however as our review highlighted, it is also possible that because of stigma and socio-cultural norms, people are reluctant to seek treatment or CHW do not recognise abuse [48]. This echoes findings of a recent systematic review which found that contextual elements such as sociocultural factors influenced CHWs performance [54]. While CHWs have been shown to be beneficial in improving uptake of care, quality of life and retention in care for other socially stigmatising conditions such as HIV [29], a study in Uganda found that due to a desire to keep their condition confidential, people living with HIV preferred CHWs who were from a different village [55].

Although not explored in any studies in this review, CHWs could play an important role in increasing access and adherence to HIV post-exposure prophylaxis (HIV PEP) as well as other treatment for survivors such as emergency contraceptive. CHWs have been used to deliver antiretroviral treatment in Uganda and Kenya for HIV-positive people at home thus making treatment more accessible and affordable [38]. In Madagascar, CHWs routinely provide contraceptives and can effectively perform pregnancy tests to inform their decision on contraceptive prescription [56]. Furthermore, a study in South Africa found that providing proactive follow up with flexible follow up locations such as the survivor’s home achieved high follow up and HIV PEP completion rates [23]. However, the authors caution that this intervention involved investing substantial resources, particularly the use of trained nurses. Community health workers, with adequate training and clarity of roles, could be a viable substitute to trained nurses in providing this type of proactive follow up in resource-constrained settings.

Our review found that only three of the seven studies included information on socio-demographic characteristics of CHWs and none included information on how these factors may have contributed to outcomes. A systematic review on factors influencing CHWs’ performance found that CHWs’ characteristics such as a high education level, experience with health condition, social status and supervision are crucial determinants of CHWs performance [57]. In addition, CHWs’ socio-demographic characteristics such as age, gender and marital status may influence clients’ perception of CHWs’ performance of specific tasks, quality of service and likelihood of dropping out. Given the highly gendered nature of sexual violence, these factors are likely to be relevant not only in terms of performance but also acceptability of CHWs services to survivors. Future studies should not only explore the effect of these socio-demographic characteristics on the acceptability of CHWs to survivors but also their effect on outcomes such as stigma, CHWs’ attitudes, CHWs’ willingness to offer sexual violence services and CHWs’ accessibility and availability.

The only programme that reported ease of delivery and minimal costs in programme had very specialised volunteers delivering a specific training intervention to a very small group. This is unlike typical CHWs programmes where CHWs have multiple responsibilities and often have to dedicate more time to their different roles. Some of the reviewed studies reported challenges that impact on the feasibility of CHWs services to survivors including long travel distances, too many responsibilities, lack of recognition and other commitments. These challenges, along with others identified in various studies, are systemic challenges which are not unique to sexual violence but affect CHWs in general, and need to be addressed to improve effectiveness of CHWs [29, 31, 58].

Lastly, our review included studies from low and middle-income countries (LMICs) as well as high-income countries. This was necessary given the limited number of studies available and the exploratory nature of our review. Nevertheless, we recognise that the available resources may influence the type of CHWs and the services they offer to survivors in the different settings. Although the information provided could not allow us to critically evaluate the differences in the models of CHWs in high-income countries compared to LMICs there were a few notable differences. The data suggest that CHWs in high-income countries were more likely to be trained for a specific intervention, attached to a specific sexual violence programme and responsible for fewer survivors [47, 50, 51]. In contrast, CHWs from LMICs were more likely to be general CHWs with sexual violence services as just one of the many healthcare services they provided [49, 52]. Thus, appropriate models of CHWs may differ depending on the resources available and more research is needed to establish the models that work for specific contexts.

### Implications and recommendations for future research

In line with current roles of CHWs, activities that CHWs could be involved in span the whole spectrum of care from primary to tertiary prevention with CHWs carrying out activities to reduce stigma and increase social support at the community level, supportive counselling, providing linkages and referral to services, supporting adherence to treatment and retention in care. A major challenge in the current review was lack of uniformity and measureable outcomes to allow comparability across different studies. Studies need to explore further various indicators of acceptability and feasibility including willingness to use, satisfaction with services, ease of delivery, quality and uptake of services, availability of resources, adequacy of training and cost-effectiveness of services. Measureable outcomes that could be assessed in studies evaluating the performance of CHWs for sexual violence care could include proportion of survivors receiving treatment within the recommended 72 h, completion of the 28 days HIV PEP and acute mental health outcomes such anxiety. Long-term outcomes include mental health complications such as depression and post-traumatic stress, STIs e.g. HIV, access to other services e.g. legal and justice, and effects on work or education.

Furthermore, it is important to consider the model of care (community and facility based, or just community), and the type of services to be offered as these will have an impact on the availability of resources and the type of training required. While the current studies reported both models and a variety of services, there was no assessment on the effectiveness of either models or the training and resources required.

Lastly, future studies should assess acceptability, feasibility and specific services for different forms of sexual violence (stranger versus intimate partners/known perpetrators) as well as age (children versus adults) as these may influence the programme design. A notable weakness in the available literature is the limited number of survivors or CHWs interviewed. To design effective CHWs programmes in sexual violence, there is need to engage more survivors and robustly monitor outcomes in CHW-led interventions. Longitudinal studies following up survivors, both with and without CHWs intervention, with clearly defined measureable outcomes can provide useful comparative data.

## Conclusions

This review points to a potential for CHWs providing support healthcare services for sexual violence but there is lack of quality evidence on appropriate models, acceptability of the services to survivors and feasibility of delivering the services. Improving services for sexual violence survivors through CHWs is only possible if acceptable and feasible models of care can be established. Overall, the studies reviewed were not designed to measure the effectiveness of CHWs services for sexual violence. Further research to establish survivor’s views on these services, and the effectiveness of these services, is crucially needed.

## Additional file

Additional file 1:
**Appendix 1.** Database Search Strategy. **Appendix 2.** Methodological quality rating of quantitative studies. **Appendix 3.** Methodological quality assessment for qualitative studies. (DOCX 57 kb)

## Abbreviations

AIDS: Acquired Immune Deficiency Syndrome
CHW: Community Health Worker
CSA: Child Sexual Abuse
EPHPP: Effective Public Health Practice Project
HIV: Human Immunodeficiency Virus
NGO: Non-governmental Organisation
NICE: National Institute for Health and Clinical Excellence
PEP: Post Exposure Prophylaxis
PRISMA: Preferred Reporting Items for Systematic reviews and Meta-Analyses
PROSPERO: International Prospective Register of Systematic Reviews
SV: Sexual Violence
TB: Tuberculosis
WHO: World Health Organisation
