# Supplementary material for: Should community health workers offer support healthcare services to survivors of sexual violence? a systematic review
Source: BMC Int Health Hum Rights. 2017 Oct 12;17:28. doi: 10.1186/s12914-017-0137-z (PMC5639742; doi:10.1186/s12914-017-0137-z)
Supplement: Additional file 1: — Appendix 1. Database Search Strategy. Appendix 2. Methodological quality rating of quantitative studies. Appendix 3. Methodological quality assessment for qualitative studies. (DOCX 57 kb) [file 12914_2017_137_MOESM1_ESM.docx]

Additional file 1

Appendix 1: Database Search Strategy

| Database |  | Domain |  | Search terms | No. of studies |
| --- | --- | --- | --- | --- | --- |
|  |  | | | | |
| Ovid MEDLINE | **1** | **Community health worker** | Keyword search | community health worker* or CHW* or lay health worker* or community own resource person* or CORP* or patient advocate* or close to community health worker* or community health aide* or village health worker* | **124,669** |
|  | **2** |  | Subject heading search | Exp community health workers/ | **3,608** |
|  | **3** | **1 or 2** |  |  | **124,669** |
|  | **4** | **Sexual violence** | Keyword search | Sexual violence or sexual abuse or sexual molestation or sexual assault or rape or date rape or defilement or incest or sodomy or child sexual abuse or post exposure prophylaxis or PEP or ARV* or antiretroviral* or HIV PEP or nPEP or n-PEP | **80,492** |
|  | **5** |  | Subject heading search | exp sexual offenses/ or exp child abuse, sexual/ or exp rape/ or exp Anti-Retroviral Agents/ or exp Antiretroviral Therapy, Highly Active/ or exp Anti-HIV Agents/ or exp Post-Exposure Prophylaxis/ | **91,886** |
|  | **6** | **4 or 5** |  |  | **124,473** |
|  | **7** | **3 and 6** |  |  | **404** |
| Africa Wide Information |  | | | | |
|  | **1** | **Community health worker** | Keyword search | community health worker* or CHW* or lay health worker* or community own resource person* or CORP* or patient advocate* or close to community health worker* or community health aide* or village health worker* | **65,041** |
|  | **2** | **Sexual violence** | Keyword search | Sexual violence or sexual abuse or sexual molestation or sexual assault or rape or date rape or defilement or incest or sodomy or child sexual abuse or post exposure prophylaxis or PEP or ARV* or antiretroviral* or HIV PEP or nPEP or n-PEP | **25,063** |
|  | **3** | **1 and 2** |  |  | **480** |
| CINAHL Plus |  | | | | |
|  | **1** | **Community health worker** | Keyword search | community health worker* or CHW* or lay health worker* or community own resource person* or CORP* or patient advocate* or close to community health worker* or community health aide* or village health worker* | **15,431** |
|  | **2** |  | Subject heading search | (MH "Community Health Workers") | **1,516** |
|  | **3** | **1 or 2** |  |  | **15,431** |
|  | **4** | **Sexual violence** | Keyword search | Sexual violence or sexual abuse or sexual molestation or sexual assault or rape or date rape or defilement or incest or sodomy or child sexual abuse or post exposure prophylaxis or PEP or ARV* or antiretroviral* or HIV PEP or nPEP or n-PEP | **22,903** |
|  | **5** |  | Subject heading search | (MH "Child Abuse, Sexual") OR (MH "Sexual Abuse+") OR  (MH "Antiretroviral Therapy, Highly Active") OR (MH "Anti-Retroviral Agents") OR (MH "Anti-HIV Agents") OR (MH "Postexposure Follow-Up") | **19,917** |
|  | **6** | **4 or 5** |  |  | **24,412** |
|  | **7** | **3 and 6** |  |  | **87** |
| Cochrane Library |  | | | | |
|  | 1 | **Community health worker** | Keyword search | community health worker* or CHW* or lay health worker* or community own resource person* or CORP* or patient advocate* or close to community health worker* or community health aide* or village health worker* | **5,339** |
|  | 2 |  | Subject heading search | Community Health Workers | **239** |
|  | 3 | **1 or 2** |  |  | **5,339** |
|  | 4 | **Sexual violence** | Keyword search | Sexual violence or sexual abuse or sexual molestation or sexual assault or rape or date rape or defilement or incest or sodomy or child sexual abuse or post exposure prophylaxis or PEP or ARV* or antiretroviral* or HIV PEP or nPEP or n-PEP | **11,295** |
|  | 5 |  | Subject heading search (exp tree) | Child abuse, Sex offenses, Antiretroviral Therapy, Highly Active, post-exposure prophylaxis, Anti-retroviral | **5,552** |
|  | 6 | **4 or 5** |  |  | **12,785** |
|  | 7 | **3 and 6** |  |  | **168** |
| Embase |  | | | | |
|  | 1 | **Community health worker** | Keyword search | community health worker* or CHW* or lay health worker* or community own resource person* or CORP* or patient advocate* or close to community health worker* or community health aide* or village health worker* | **205,085** |
|  | 2 |  | Subject heading search | Exp community care/ or exp health auxiliary/ | **107,048** |
|  | 3 | **1 or 2** |  |  | **309,946** |
|  | 4 | **Sexual violence** | Keyword search | Sexual violence or sexual abuse or sexual molestation or sexual assault or rape or date rape or defilement or incest or sodomy or child sexual abuse or post exposure prophylaxis or PEP or ARV* or antiretroviral* or HIV PEP or nPEP or n-PEP | **120,249** |
|  | 5 |  | Subject heading search | exp sexual abuse/ or exp sexual violence/ or exp rape/ or exp highly active antiretroviral therapy/ or exp antiretrovirus agent/ or exp post exposure prophylaxis | **176,975** |
|  | 6 | **4 or 5** |  |  | **223,619** |
|  | 7 | **3 and 6** |  |  | **2,548** |
| Global Health |  | | | | |
|  | 1 | **Community health worker** | Keyword search | community health worker* or CHW* or lay health worker* or community own resource person* or CORP* or patient advocate* or close to community health worker* or community health aide* or village health worker* | **18,472** |
|  | 2 |  | Subject heading search | (community health or community health services).sh. | **5,948** |
|  | 3 | **1 or 2** |  |  | **23,721** |
|  | 4 | **Sexual violence** | Keyword search | Sexual violence or sexual abuse or sexual molestation or sexual assault or rape or date rape or defilement or incest or sodomy or child sexual abuse or post exposure prophylaxis or PEP or ARV* or antiretroviral* or HIV PEP or nPEP or n-PEP | **40,188** |
|  | 5 |  | Subject heading search | ("rape (trauma)" or sexual abuse or antiretroviral agents or highly active antiretroviral therapy).sh. | **22,380** |
|  | 6 | **4 or 5** |  |  | **40,188** |
|  | 7 | **3 and 6** |  |  | **233** |
| PSYCHInfo |  | | | | |
|  | 1 | **Community health worker** | Keyword search | community health worker* or CHW* or lay health worker* or community own resource person* or CORP* or patient advocate* or close to community health worker* or community health aide* or village health worker* | **36,759** |
|  | 2 |  | Subject heading search | exp Community Services/ | **27,122** |
|  | 3 | **1 or 2** |  |  | **63,473** |
|  | 4 | **Sexual violence** | Keyword search | Sexual violence or sexual abuse or sexual molestation or sexual assault or rape or date rape or defilement or incest or sodomy or child sexual abuse or post exposure prophylaxis or PEP or ARV* or antiretroviral* or HIV PEP or nPEP or n-PEP | **41,043** |
|  | 5 |  | Subject heading search | exp Sexual Abuse/ or exp Sex Offenses/ or exp Rape/ or exp Antiviral Drugs/ | **31,424** |
|  | 6 | **4 or 5** |  |  | **45,771** |
|  | **7** | **3 and 6** |  |  | **697** |
|  |  |  |  | **Total (all row 7’s)** | **4,617** |

Appendix 2: Methodological quality rating of quantitative studies

1. Quality rating pre-post test study (EPHPP checklist)

| **Rating domain** | **Rating scale** | **Barron, 2013 [**[**47**](#_ENREF_47)**]** |
| --- | --- | --- |
| **A) Selection bias** | |  |
| Are the individuals selected to participate in the study likely to be representative of the target population? |  | Not likely |
| What percentage of selected individuals agreed to participate? |  | 100% |
| **Section rating** | Strong, Moderate or Weak | Moderate |
| **B) Study design** | |  |
| Indicate the study design  Was the study described as randomized? No/Yes  If Yes, was the method of randomization described? No/Yes  If Yes, was the method appropriate? No/Yes |  | Pre- posttest waitlist  No |
| **Section rating** | Strong, Moderate or Weak | Moderate |
| **C) Confounders** | |  |
| Were there important differences between groups prior to the intervention? |  | Yes: Sex, age |
| If yes, indicate the percentage of relevant confounders that were controlled (either in the design (e.g. stratification, matching) or analysis)? |  | Can’t tell |
| **Section rating** | Strong, Moderate or Weak | Weak |
| **D) Blinding** | |  |
| Was (were) the outcome assessor(s) aware of the intervention or exposure status of participants? |  | Yes |
| Were the study participants aware of the research question? |  | Can’t tell |
| **Section rating** | Strong, Moderate or Weak | Weak |
| **E) Data collection methods** | |  |
| Were data collection tools shown to be valid? |  | Yes |
| Were data collection tools shown to be reliable? |  | Can’t tell |
| **Section rating** | Strong, Moderate or Weak | Moderate |
| **F) Withdrawals and dropouts** | |  |
| Were withdrawals and drop-outs reported in terms of numbers and/or reasons per group? |  | Yes |
| Indicate the percentage of participants completing the study. (If the percentage differs by groups, record the lowest). |  | 100% |
| **Section rating** | Strong, Moderate or Weak | Strong |
| **G) Intervention integrity** | |  |
| What percentage of participants received the allocated intervention or exposure of interest? |  | 100% |
| Was the consistency of the intervention measured? |  | Yes |
| Is it likely that subjects received an unintended intervention (contamination or co-intervention) that may influence the results? |  | Can’t tell |
| **Section rating** | Strong, Moderate or Weak | Moderate |
| **H) Analyses** | |  |
| Indicate the unit of allocation |  | Individual |
| Indicate the unit of analysis |  | Individual |
| Are the statistical methods appropriate for the study design? |  | Yes |
| Is the analysis performed by intervention allocation status (i.e. intention to treat) rather than the actual intervention received? |  | Can’t tell |
| **Section rating** | Strong, Moderate or Weak | Moderate |

1. Quality rating of longitudinal study (QATSO Scale)

|  | **Domain** | | **Scoring** | **Kohli, 2012 [**[**49**](#_ENREF_49)**]** |
| --- | --- | --- | --- | --- |
| 1 | Was the sampling method representative of the population intended to the study? | A. Non-probability sampling (including: purposive, quota , convenience and snowball sampling) | 0 | **√** |
|  |  | B. Probability sampling (including: simple random, systematic, stratified g, cluster, two-stage and multi-stage sampling) | 1 |  |
| 2 | Was the measurement of outcome objective)? | By questionnaires (Self-reported) | 0 | **√** |
|  |  | By clinical records or lab tests | 1 | **√** |
|  |  | Not applicable | NA |  |
| 3 | Did the study report any response rate? | No | 0 |  |
|  |  | Yes | 1 |  |
|  |  | Not applicable | NA | **√** |
| 4 | Did the investigator(s) control for confounding factors (e.g. stratification/ matching/ restriction/ adjustment) when analysing the associations (if the study contains purely descriptive results, no association and prediction tests were conducted in the test, please select “Not applicable”)? | No | 0 |  |
|  |  | Yes | 1 |  |
|  |  | Not applicable | NA | **√** |
| 5 | Was privacy or sensitivity of the nature of condition (sexual violence) considered when the survey was conducted e.g. if conducted in a general clinic setting? | No | 0 |  |
|  |  | Yes | 1 | **√** |

Scoring method: Total score divided by total number of all applicable items

Grading of the QATSO score:

| 0% -33% | 33%- 66% | 67% -100% |
| --- | --- | --- |
| Bad | Satisfactory | Good |

Appendix 3: Methodological quality assessment for qualitative studies

|  |  | Barron, 2013 [[47](#_ENREF_47)] | Itzhaky, 2001 [[48](#_ENREF_48)] | Merkin, 1995 [[50](#_ENREF_50)] | Rossman, 1999 [[51](#_ENREF_51)] | Tanabe, 2013 [[52](#_ENREF_52)] | Zraly, 2011 [[53](#_ENREF_53)] |
| --- | --- | --- | --- | --- | --- | --- | --- |
| Section 1: theoretical approach  1.1 Is a qualitative approach appropriate? | Appropriate | **√** | **√** | **√** | **√** | **√** | **√** |
|  | Inappropriate |  |  |  |  |  |  |
|  | Not sure |  |  |  |  |  |  |
| 1.2 Is the study clear in what it seeks to do? | Clear | **√** |  |  |  | **√** |  |
|  | Unclear |  |  |  | **√** |  |  |
|  | Mixed |  | **√** | **√** |  |  | **√** |
| Section 2: study design  2.1 How defensible/rigorous is the research design/methodology? | Defensible | **√** | **√** |  |  | **√** | **√** |
|  | Not defensible |  |  | **√** | **√** |  |  |
|  | Not sure |  |  |  |  |  |  |
| Section 3: data collection  3.1 How well was the data collection carried out? | Appropriate | **√** | **√** |  |  | **√** | **√** |
|  | Inappropriate |  |  |  |  |  |  |
|  | Not sure/ inadequately reported |  |  | **√** | **√** |  |  |
| Section 4: validity  4.1 Is the context clearly described? | Clear | **√** | **√** |  |  | **√** | **√** |
|  | Unclear |  |  | **√** | **√** |  |  |
|  | Not sure |  |  |  |  |  |  |
| 4.2 Were the methods reliable? | Reliable | **√** | **√** |  |  |  |  |
|  | Unreliable |  |  | **√** | **√** | **√** | **√** |
|  | Not sure |  |  |  |  |  |  |
| Section 5: analysis  5.1 Are the data 'rich'? | Rich | **√** |  |  |  | **√** |  |
|  | Poor |  |  | **√** |  |  |  |
|  | Not sure/not reported |  | **√** |  | **√** |  | **√** |
| 5.2 Is the analysis reliable? | Reliable |  |  |  |  |  |  |
|  | Unreliable |  |  |  |  | **√** | **√** |
|  | Not sure/not reported | **√** | **√** | **√** | **√** |  |  |
| 5.3 Are the findings convincing? | Convincing | **√** |  |  |  | **√** | **√** |
|  | Not convincing |  | **√** | **√** | **√** |  |  |
|  | Not sure |  |  |  |  |  |  |
| 5.4 Are the conclusions adequate? | Adequate | **√** | **√** | **√** | **√** | **√** | **√** |
|  | Inadequate |  |  |  |  |  |  |
|  | Not sure |  |  |  |  |  |  |
| Section 6: ethics  6.1 Was the study approved by an ethics committee? | Yes | **√** |  |  |  | **√** | **√** |
|  | No |  |  |  |  |  |  |
|  | Not sure/not reported/ not applicable |  | **√** | **√** | **√** |  |  |
| 6.2 Is the role of the researcher clearly described? | Clear | **√** |  |  |  | **√** | **√** |
|  | Not clear |  | **√** | **√** |  |  |  |
|  | Not sure/not reported |  |  |  | **√** |  |  |
| Section 7: Overall assessment  As far as can be ascertained from the paper, how well was the study conducted? | ++ | **√** |  |  |  | **√** | **√** |
|  | + |  | **√** |  |  |  |  |
|  | − |  |  | **√** | **√** |  |  |
